# Electroactive Microorganisms in Advanced Energy Technologies

**DOI:** 10.3390/molecules28114372

**Published:** 2023-05-26

**Authors:** Xingchen Zhou, Xianzheng Zhang, Yujie Peng, Abdoulkader Ibro Douka, Feng You, Junlong Yao, Xueliang Jiang, Ruofei Hu, Huan Yang

**Affiliations:** 1 Hubei Key Laboratory of Plasma Chemistry and Advanced Materials, School of Materials Science and Engineering, Key Laboratory of Green Chemical Engineering Process of Ministry of Education, Wuhan Institute of Technology, No. 206 Guanggu 1st Road, Wuhan 430205, China; zhouxingchen_123@163.com (X.Z.); zhangxianzheng1122@163.com (X.Z.); meraki.plin@foxmail.com (Y.P.); youfeng.mse@wit.edu.cn (F.Y.); jlyao@wit.edu.cn (J.Y.); jiangxl@wit.edu.cn (X.J.); 2 State Key Laboratory of Fine Chemicals, Zhang Dayu School of Chemistry, Dalian University of Technology, Dalian 116024, China; akimdouka@dlut.edu.cn; 3 Department of Food Science and Chemical Engineering, Hubei University of Arts and Science, Xiangyang 441053, China

**Keywords:** microorganisms, electrocatalysis systems, microbial sensors, microbial power generation

## Abstract

Large-scale production of green and pollution-free materials is crucial for deploying sustainable clean energy. Currently, the fabrication of traditional energy materials involves complex technological conditions and high costs, which significantly limits their broad application in the industry. Microorganisms involved in energy production have the advantages of inexpensive production and safe process and can minimize the problem of chemical reagents in environmental pollution. This paper reviews the mechanisms of electron transport, redox, metabolism, structure, and composition of electroactive microorganisms in synthesizing energy materials. It then discusses and summarizes the applications of microbial energy materials in electrocatalytic systems, sensors, and power generation devices. Lastly, the research progress and existing challenges for electroactive microorganisms in the energy and environment sectors described herein provide a theoretical basis for exploring the future application of electroactive microorganisms in energy materials.

## 1. Introduction

At present, large-scale exploitation of fossil energy has caused severe persecution on our environment and climate over the last few decades [1,2,3,4,5,6,7,8]. High-efficiency, environmentally friendly, and low-cost energy materials have attracted wide attention for applications in material science, biomedical science, and environmental science [9,10,11,12,13,14]. Large-scale production of promising green and sustainable materials is vital to convert renewable energy sources efficiently. Recently, advanced energy materials in electrocatalysis, biosensing, fuel cells, and power generation have been widely studied [11,15,16,17,18,19]. However, preparing traditional energy materials still involves complex technological conditions and high preparation costs, which dramatically limit the large-scale application of energy materials in the industry.

In nature, microorganisms widely exist in nature under various extreme environments. Generally, a large number of microorganisms can lead to the spread of infectious diseases, food decay, and metal corrosion. Electroactive microorganisms have a diversity of functions in anaerobic soil, intestinal systems, sediment, and other environments, where they can have electrical interactions with the environment. Microorganisms possess the advantages of simple morphological structures, such as balls, rods, screws, and branched filaments, which can be used as natural biological templates to construct energy materials [20,21,22,23]. Microbial cells with rich metal and nonmetallic elements can be used as elemental doping sources [24]. This method simplifies the steps in the synthetic process and enables the sustainable development of green energy materials. More interestingly, the microorganisms have various oxidoreductases [25,26]. This biological redox protein can catalyze the electron transfer reaction by reducing/oxidizing substrates [27]. The electrical signals produced from electron transfer can be widely used in electrosynthesis, sensors, and electrocatalysis [28,29,30]. In addition, the microbial metabolic process can convert respiratory substrates into substances, such as proteins, organic acids, sugars, and vitamins, realizing bidirectional electron transfer and energy exchange with the external environment [31,32].

In recent years, microbial electrochemical technology has demonstrated vast application potential in electric power production, environmental remediation, biosensing, and other fields [33]. This technology has been employed in microbial fermentation for hydrogen production, degradation of organic pollutants, wastewater treatment, etc. Microbial electrochemical systems involve complex mechanisms [34]. Many studies have been conducted to realize various electrochemical systems through extracellular electron transport and energy transfer between electroactive microorganisms and electrodes. The mechanisms of microorganism action in electrochemical systems are summarized in several recent reports, and we do not examine them in detail here [35,36]. Instead, we focus on the influence of microbial characteristics on the efficiency of electrochemical systems and the ways to regulate them [37]. The low extracellular electron transport efficiency of traditional electroactive microorganisms leads to the limited energy density of electrochemical systems, impeding their practical application. Regulating the structure and composition of electrode biofilms, the growth of biofilms, and the electron transfer mechanisms between microorganisms and electrodes is essential to realize efficient electrochemical reactions.

This review illustrates the advantages of electroactive microorganisms in electron transfer, growth metabolism, redox, and structural and compositional properties. The critical factors of electroactive microorganisms toward advanced energy materials are explored from the relationship between biological characteristics and electrochemical performance. The applications of microbial characteristics in energy materials, including electrocatalytic materials, biosensors, and power generation devices, are classified and evaluated. Finally, the development prospects for electroactive microorganisms and challenges in energy materials applications are proposed.

## 2. Electrocatalytic Systems

In general, microbial characteristics such as electron transfer, rich structure, and composition can be applied to construct advanced electrocatalytic materials in electrocatalytic systems [38,39,40]. The composition, crystal surface, size, and morphology of the electrode surface are important factors affecting the electrocatalytic system’s performance. Compared with conventional electrocatalytic materials, biomass-based electrode surfaces have the merits of large active sites and defects, controllable composition, and superior structure, which can be applied in electrocatalytic systems [41,42,43,44,45,46,47].

### 2.1. Microbial Properties in Electrocatalytic Systems

**Figure 1:**
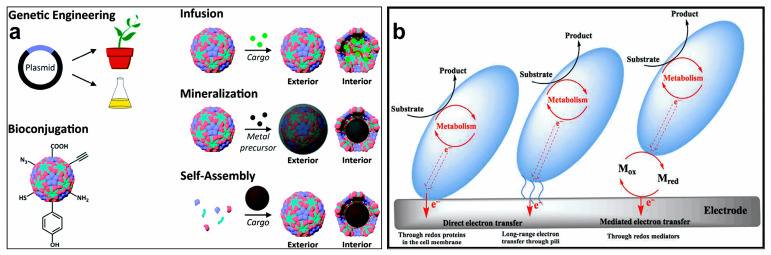
(**a**) Common methods for the internal and external modification of viruses. Reprinted with permission [48]. Copyright 2016, Royal Society of Chemistry. (**b**) Three mechanisms of electron transfer in microbial cells. Reprinted with permission [54]. Copyright 2019, Elsevier.

In nature, microorganisms have abundant structural configurations, including spherical, rod, spiral, or branched filaments. Their surface contains various organic functional groups, presenting rich sites for various reactions. The diversity of morphologies can act as natural biological templates for electrocatalysts. Genetic engineering, bioconjugation, infusion, and biological assembly of viruses are effective strategies for preparing efficient functional nanocatalyst materials (Figure 1a) [48]. Viruses (such as *filamentous M13 bacteriophage*) can be genetically engineered to produce a variety of virus-based and virus-like particles with excellent properties. Owing to their unique shell structure, residues (and functional groups) can be inserted or replaced to functionalize viruses with gene-encoded proteins. Genetic engineering can be introduced to directionally modify materials by regulating their composition and microstructure [49]. The genetic engineering technology of microorganisms can also load and immobilize nanomaterials, which can be scaled up to produce a variety of recyclable electrocatalytic systems. For example, *Escherichia coli* (*E. coli*) has been genetically modified to be photosensitive; the secreted histidine-tagged protein fibers under light conditions can be loaded and immobilized on the biofilm fiber network through the metal coordination, thereby reducing the damage to cells caused by the catalysts. This microbial genetic engineering technology provides a new scheme for hydrogen production and catalytic degradation [50,51]. The variety of functional groups attached at the surface of the subunits, membranes, and biopolymers can adsorb metal ions and rapidly reproduce; thus, microbes can be used to synthesize nanomaterials. In addition to the diverse structure of microorganisms, nonmetallic elements, such as C, N, P, and S, exist in microbial cells, and various metal elements are also enriched in the microbial metabolic process. These active elements in the cells act as a heteroatom source and can be doped into heterogeneous electrocatalysts to produce many active sites and defects, providing an efficient and straightforward strategy to prepare electrocatalysts [52,53].

Extracellular electron transport is divided into direct electron transfer (redox proteins, nanowires) and indirect electron transfer (diffusible redox mediators). These two mechanisms have been proposed to elucidate the respiratory activities of electroactive microbial substrates (Figure 1b) [41,54,55]. Generally, an electron transition can proceed by the microbial membrane and the extracellular acceptors. Direct electron transfer refers to direct physical contact between extracellular electron acceptors and redox proteins on the membrane surface. In addition, conductive pili on the microbial surface can be used to form microbial nanowires, enabling direct electron transfer over long distances and increasing the chances of microbial interactions with extracellular electron acceptors. Indirect electron transfer uses diffusible redox mediators as electron carriers to transport the released or received electrons, breaking the distance limit between microorganisms and extracellular electron acceptors, and completing the electron transfer between microorganisms and extracellular electron acceptors. The electroactive microorganisms can induce the redox potential difference between the metabolic-mediated electrode and the microorganism, which can improve the internal and external electron transfer efficiency of the electrode [56]. This electron transfer rate is a critical factor in affecting the reaction dynamics of the microbial electrocatalytic system. The adsorption and diffusion of microorganisms on the solid surface can significantly affect their electron transport efficiency, which is closely related to the amount of charge, surface area, porosity, thickness, and biocompatibility of the microorganism membrane interface. The microorganisms’ metabolism can transform the intracellular substances and other foreign molecules into proteins, nucleic acids, fat, and other substances [57]. These abundant metabolites and extremely high metabolic rates contribute to microorganisms’ highly efficient biological properties [58,59,60]. More importantly, the active substances and functional groups on the microorganisms can reassemble metal ions into nanocrystal particles, a process that is beneficial for constructing electrode materials with high catalytic activity [61].

### 2.2. Electroactive Microorganisms in Electrocatalysis

In the electrocatalytic system, the electron transfer characteristics of electroactive microorganisms couple microbial metabolism and electrode catalysis to achieve more efficient electrocatalytic reaction kinetics. The efficient electrocatalytic reaction kinetics enables clean energy production and organic waste treatment [52]. The application of microorganisms in electrocatalysts involves hydrogen evolution reaction (HER), oxygen evolution reaction (OER), oxygen reduction reaction (ORR), carbon dioxide reduction reaction (CO_2_RR), etc. These multistep reaction processes involve various intermediates, such as H*, HO*, COOH*, CO*, and CH_3_O* [62]. The adsorption/desorption of reaction intermediates on the catalyst surface directly affect their catalytic activity. In general, regulating the structure and vacancy of the surface, including substituting exogenous elements, phase transition, and strain, are effective strategies to increase the intrinsic activity or active sites of electrocatalysts. Extracellular electron transfer, unique structure, and composition of microorganisms can be used as biological templates and heteroatom sources to construct efficient electrocatalysts [20,63].

**Figure 2:**
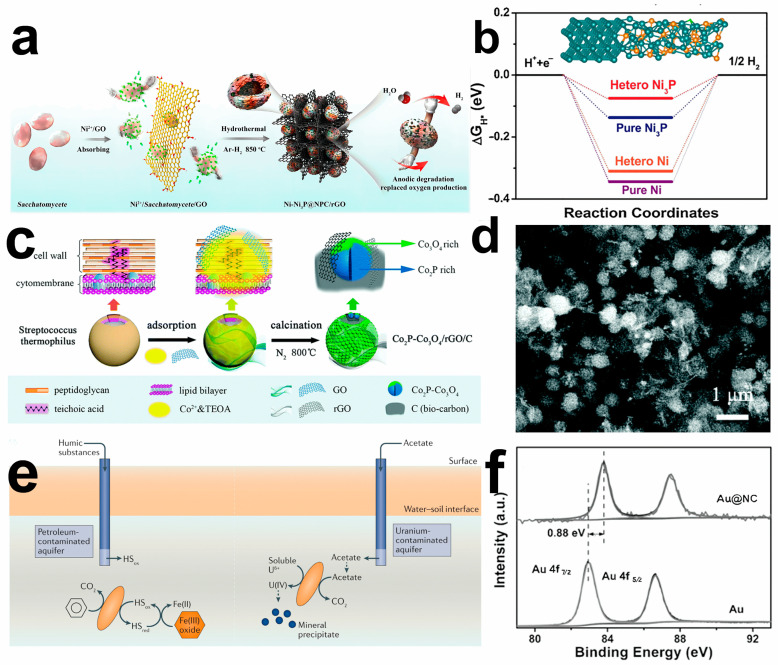
(**a**) Schematic illustration for the preparation and application of Ni-Ni_3_P@NPC/rGO. (**b**) Free energy diagram of different samples for HER. Reprinted with permission [64]. Copyright 2020, Elsevier. Schematic diagram for the fabrication process (**c**) and field emission scanning electron microscopy images (**d**) of Co_2_P-Co_3_O_4_/rGO/C. Reprinted with permission [65]. Copyright 2020, Royal Society of Chemistry. (**e**) Schematic illustration for the growth and reduction of electroactive microorganisms. Reprinted with permission [62]. Copyright 2022, Springer Nature. (**f**) X-ray photoelectron spectroscopy survey spectrum of Au in Au@NC. Reprinted with permission [66]. Copyright 2016, Wiley.

For instance, yeast cells possess globular porous structures and rich N and P contents in organisms. Yeast cells can adsorb Ni ions to the cell wall and serve as biological carbon templates [64]. After hydrothermal reaction and annealing, reduced graphene oxide (rGO) sheets are uniformly distributed. The Ni-Ni_3_P nanoparticles are embedded into N, P codoped carbon (NPC) on 3D graphene frameworks (Ni-Ni_3_P@NPC/rGO) (Figure 2a). Among them, N and P embedded in carbon-based templates and the heterostructure of Ni-Ni_3_P are beneficial to reduce the hydrogen adsorption during the HER process, improving the catalytic activity (Figure 2b).

Moreover, yeast’s porous and component-rich properties can also be applied to OER electrocatalysts. RuO_2_ is the most active OER catalyst in acidic electrolytes, with a relatively small starting potential and exceptionally superior stability. The yeast surface-adsorbed Ru ion becomes a dense and hard RuO_2_ layer that may form NPC@RuO_2_ in a quasi-vacuum environment by further pyrolysis. The resulting NPC@RuO_2_ of the yolk-shell structure, with an overpotential of 220 mV, reaches a current density of 10 mA cm^−2^ in 0.5 M sulfuric acid [67]. The cell wall of Gram-positive bacteria contains teichoic acid and peptidoglycan [65]. The abundant functional groups can adsorb Co ion on the surface of Gram-positive bacteria, and the release of organic matter can be transferred into oxide, forming metal-rich phosphide and abundant oxide surface. To further improve electron transport efficiency, GO and Co ions are added to modify the cell wall. Co_2_P-Co_3_O_4_ nanoparticles and rGO sheets obtained by calcination are uniformly distributed on submicron-spherical C substrates (Figure 2c). The internal substructure and gap between Co_2_P-Co_3_O_4_ nanoparticles and rGO sheets are beneficial for forming a porous structure with high specific surface area (Figure 2d). The as-obtained Co_2_P-Co_3_O_4_/rGO/C composite shows excellent electrocatalytic performance for ORR with an onset and half-wave potential of 0.91 V vs. reversible hydrogen electrode (RHE) and 0.80 V vs. RHE, respectively.

In addition, electroactive microorganisms can be applied to biosynthesize excellent electrocatalysts through corrosion and reduction (Figure 2e) [68,69]. Interestingly, the *Pycnoporus sanguineus* cells can reduce Au^3+^, and the Au nanoparticles supported on N-doped carbon (Au@NC) core-shell structure is synthesized by calcination [66]. Au nanoparticles formed on the carbon carrier and electrons can transfer from the Au to the NC shell, which induces its positive shift of binding energy against pure gold nanoparticles, significantly influencing the catalytic activity of Au@NC (Figure 2f). Au@NC has highly efficient HER activity with a small onset potential of only 54.1 mV vs. RHE and a Tafel slope of 76.8 mV dec^−1^.

Electrochemical CO_2_ reduction technology is a new energy storage method to convert electrical energy into chemical energy, but it currently needs to be improved by low activity and poor stability [70]. The design of efficient and rational CO_2_RR catalysts is essential for producing renewable and sustainable fuels. *Sporomusa ovata* has been genetically engineered to improve its methanol adaptability [71]. Autotrophic metabolism of *Sporomusa ovata* was accelerated using methanol as an electron donor. Culturing methanol-adapted *Sporomusa ovata* on silicon nanowires improved the catalytic efficiency of CO_2_RR by enhancing microbial metabolic activity. The synergy of the high-surface-area cathode and the adapted strain achieves a CO_2_-reducing current density of 0.88 ± 0.11 mA cm^−2^.

## 3. Microbial Sensors

### 3.1. Microbial Properties in Microbial Sensors

**Figure 3:**
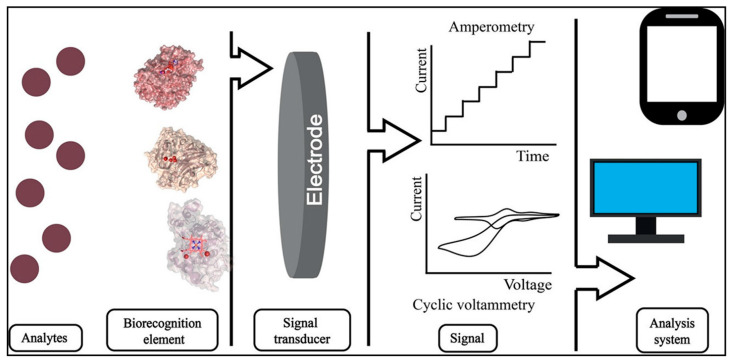
A scheme of electrochemical enzymatic biosensors. Reprinted with permission [40]. Copyright 2020, American Chemical Society.

Oxidoreductases are biological redox proteins in microbial cells and can catalyze electron transfer reactions by reducing or oxidizing substrates in the biosensing field. When the microorganism anchors to the electrode surface, the enzyme’s active site binds to the recipient electrode (Figure 3) [40]. This electron transfer of redox reactions at the interface can produce different electrical signals, which presents great potential for applications in electrochemical microbial sensors. In general, redox reactions occur at the enzyme electrode–electrolyte interface, and the electron transfer rate at the interface is the critical factor affecting microbial sensors’ performance. Electrons can be directly transferred from the electrode to the enzyme’s active site for microorganisms with proteins, ferredoxin, and peroxidase [72]. This direct electron transfer mechanism requires a distinct active site and a short distance between the active site and electrode surface of the enzyme. Only a tiny fraction of proteins can undergo direct electron transfer in redox-transformed electrochemical systems [73]. When the enzyme’s active site in most microbial bodies is not obvious, or the distance between the active site and the electrode surface is considerable, the redox mediators (flavins, quinones) are required to shuttle the electrons between the enzyme and the electrode [74]. For example, *Pseudomonas aeruginosa* can generate various phenazine metabolites with redox activity. These phenazine metabolites act as electron shuttles to directly transfer electrons [75,76].

When the oxidoreductases are fixed on the electrode surface, the redox cofactors of microorganisms and the electrode potential can affect the biosensing reaction dynamics [77,78]. In general, the redox potential of the mediator should be within the catalytic potential of the enzyme, and a sufficient potential difference is required to provide the thermodynamic driving force [79]. Therefore, the critical factors, including specificity of the enzyme, the redox cofactor motif, the diffusion ability of the mediator, and the membrane activity of the oxidoreductase at the electrode, can affect the performance of the enzymatic electrochemical system [80]. Other vital strategies, such as the addition of non-natural electronic media, the design of an efficient heterogeneous metabolic pathway, and the membrane structure of enzymes at the electrode, can be adopted to determine the reaction efficiency of microbial sensing system [81].

### 3.2. Electroactive Microorganisms in Microbial Sensors

Microbial sensors can be used as clinical diagnostic tools to rapidly detect pathogen infection, deoxyribonucleic acid, pathogens, and hormones [82,83]. Microbial sensors usually comprise immobilized microbial film, signal output, and transducer [84]. The whole microorganism cell can be directly fixed on the electrode interface to form microbial membrane, which can be used as sensitive material for molecular recognition. Microbial sensors can be widely applied in environmental detection and clinical diagnosis with the merits of small size, easy to produce, low price, and degradable characteristics [85]. Table 1 summarizes the performance of different types of microbial sensors.

**Figure 4:**
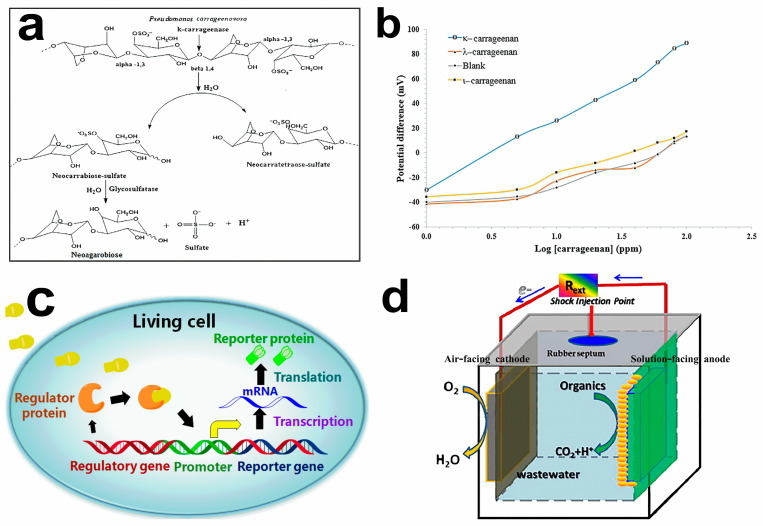
(**a**) Schematic diagram of the bioenzymatic cascade reaction of κ-carrageenan. (**b**) The kinetic curves of the potentiometric biosensor toward 1–100 ppm κ-, ι-, and λ-carrageenan by using the separate solution method. Reprinted with permission [96]. Copyright 2019, Public Library of Science. (**c**) A schematic diagram of a typical whole cell-based biosensor. Reprinted with permission [97]. Copyright 2017, MDPI. (**d**) Schematic depiction of the single chamber batch-mode cube microbial fuel cell as the shock sensor. Reprinted with permission [98]. Copyright 2014, Elsevier.

Interestingly, the outer membrane proteins and lipopolysaccharides can physically and chemically react with different types of sensors. Microorganisms do not require protein isolation or a purification step. *Pseudomonas carrageenovora* cells produce κ-carragenase and glycosulfatase for the specific determination of κ-carrageenan. The catalytic layer can be formed when the cell is fixed on the membrane electrode. The bienzymatic cascade reaction can catalyze the decomposition of κ-carrageenan. The κ-carragenase catalyzes the hydrolytic cleavage of the internal β-(1,4) glycosidic bond of the κ-carrageenan to produce neocarrabiose sulfate [3-O-(3,6-anhydro-α-D-galactopyranosyl)-D-galactopyranose-4-O-sulfate] and neocarratetraose-sulfate. The glycosulfatase catalyzes the enzymatic desulfation of neocarrabiose sulfate to neoagarobiose [3-O-(3,6-anhydro-α-L-galactopyranosyl)-D-galactopyranose], sulfate, and hydrogen ions. The concentration of κ-carrageenan was determined indirectly by the detected hydrogen ions concentration (Figure 4a,b) [96].

Currently, the widespread application of microbial sensors is still facing some difficulties. The toxic factors, such as heavy metals and toxic organic matter in the measured objects, can affect the stable response and life span of microorganisms [99,100]. Developing effective strategies, including microbial breeding, genetic engineering, and cell fusion technologies, can be used to construct mild immobilization techniques and improve the air permeability of microbial membranes, obtaining highly sensitive microbial nanosensors [101,102]. Microbial sensors are less sensitive to environmental changes than molecular-based biosensors [103]. Intact microbial living cells can be used as entities by modifying microbial genetic material. The introduced reporter gene system can operate under broader conditions, such as various temperatures and potential (Figure 4c) [97,104].

In addition to the traditional microbial sensors, devices that use energy obtained during wastewater treatment can be used as a self-powered sensor [105]. A novel microbial electrochemical device to treat wastewater was discovered using a microbial fuel cell (MFC) as a microbial sensor (Figure 4d) [98]. MFCs can directly convert wastewater into electricity by producing electrogenic bacteria. When used as a wastewater sensor, wastewater can cause a change in system voltage output, which can be monitored in situ. Without external power, the power generated through MFCs can support their operation. This novel MFC is a new biosensing device with high efficiency, low cost, and energy sustainability.

## 4. Power Generation Devices

With the development of current bioenergy technologies, electroactive microorganisms have great potential in power generation devices [47]. For example, some bacteria can break down organic molecules in the battery pack to release electrons, providing electricity externally by forming bacterial fuel cells. This process is often related to microorganism diversity, metabolic activity, and structural configuration [106,107]. More interestingly, some electroactive microorganisms can produce electric currents; their electrically conductive filaments can be used to mediate long-distance power generation [56]. Based on these microorganisms’ composition and structural characteristics, electroactive microorganisms can form positive/negative electrodes by self-assembly, heteroatomic doping, and activation methods, which can efficiently generate electricity, attracting wide attention concerning lithium batteries and air power generation fields [108,109].

### 4.1. Microbial Properties in Power Generation Devices

In microbial power generation, efficient electron transfer between electroactive microorganisms and electrodes is essential to define their performance and suitability. The electron transfer of several microorganisms can extend to the cell membrane through conductive nanowires (flagella, membrane capsule) with unique electron tracks [110]. Similar to metals, extracellular conducting nanowires have a unique electron transport mechanism. Electrons generated within cells can transport conductive nanowires to extracellular receptors, increasing the interaction between microbes and extracellular receptors, which has great potential in power generation devices. Various important factors, such as the number of surface functional groups, the porosity and pore distribution of microbial nanowire membrane, and the conductivity and network structure of the nanowire membrane–electrode interface, can significantly affect the efficiency and stability of power generation. For instance, the protein nanowires extracted from *Geobacter* species can be made into thin-film power generation devices [8]. For their composition and structural characteristics, some microorganisms contain reducing agent components, such as sodium borohydride, hydride, hydrazine, H_2_, etc., which can replace the necessary chemical reducing agent and electrode material [111].

### 4.2. Electroactive Microorganisms in Protein Nanowires

**Figure 5:**
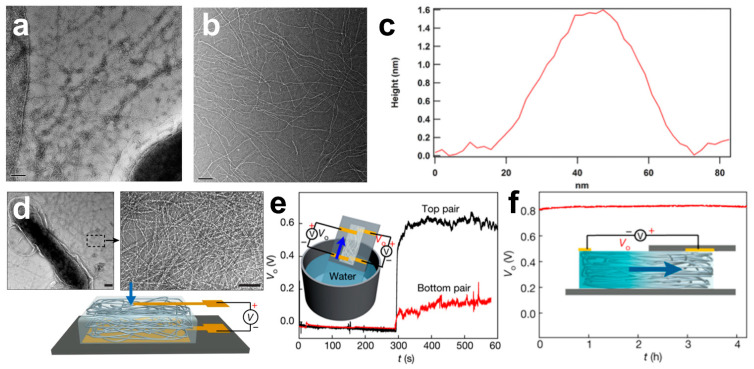
Transmission electron microscopy image of strain W51W57 (**a**) and the pili harvested from strain W51W57 (**b**) (scale bars represent 100 nm). (**c**) Height measurement of strain W51W57 pili with a white line at the indicated location. Reprinted with permission [114]. Copyright 2016, Wiley. (**d**) Transmission electron microscope images of the purified nanowire network (scale bars represent 100 nm). (**e**) V_0_ from the top and bottom pairs of electrodes after the devices are closed to the water surface. (**f**) Voltage produced by the moisture gradient between the two top electrodes. Reprinted with permission [56]. Copyright 2020, Springer Nature.

Protein nanowires have proteins with unique electronic tracks and conductive flagella [112]. They can serve as nanoscale “wires” to transport nutrients and communicate with other bacteria [113]. This biosynthesis enables the sustainable production of conductive nanowires without harsh synthesis conditions. In this complex enzymatic conducting network, the aspect ratio, the electrical conductivity, and the fixation of the conducting flagellum are the key factors limiting the enzyme’s power generation efficiency. The conductive flagella on the *Geobacter sulfurreducens* surface are assembled from the peptide monomer PilA, which contains abundant phenylalanine and tyrosine, contributing to π-π stacking along the flagellum for electron transport. Nevertheless, tryptophan can promote electron transport more effectively than phenylalanine or tyrosine. When tryptophan was added to the PilA protein by genetic engineering, the strain W51W57 with abundant pili was generated (Figure 5a,b). Owing to the excellent binding between the amino acids, the diameter of W51W57 pili is only 1.5 nm, half the diameter of the wild type, and the conductivity is increased several thousand times (Figure 5c) [114].

In addition, compared to traditional power generation technologies, air power generation, with the merits of sustainable production, small size, and low price, acts as a new clean energy technology [115]. Protein nanowires of many bacteria can transfer electrons from organic material to other bacteria or the environment in which they are located. In this regard, a thin-film device based on protein nanowires is designed to use water in the air for power generation [56]. This generator generates a continuous current for at least 20 h before self-charging with a power density of about 4 mW cm^−3^. The device consists of the bottom of a protein nanofilm produced by *Geobacter* and a gold electrode mounted on glass. Protein nanowires grown by the *Geobacter sulfurreducens* form a network structure on the membrane that can absorb water vapor from the atmosphere (Figure 5d). The moisture gradient caused by the water adsorption difference between the electrodes is an important factor in generating voltage (Figure 5e,f). The presence of these nanoholes in the film can explain the presence of a moisture gradient, and the thin pores between the film nanowires can generate a current between the two electrodes. This device, which requires only about 10 microns of protein-based nanowires, can quickly produce large numbers of protein-based nanowire thin-film devices. In this air power generation device, controlling the nanopore distribution, its structure, and the density of surficial functional groups, and managing charge diffusion, pH, and relative humidity of the solution are important strategies for improving the voltage efficiency. For instance, the modified *E. coli* can produce large amounts of the solanum pigment, allowing the bacteria attached to the electrodes to generate electricity through photosynthesis [116].

### 4.3. Electroactive Microorganisms in Lithium Batteries

**Figure 6:**
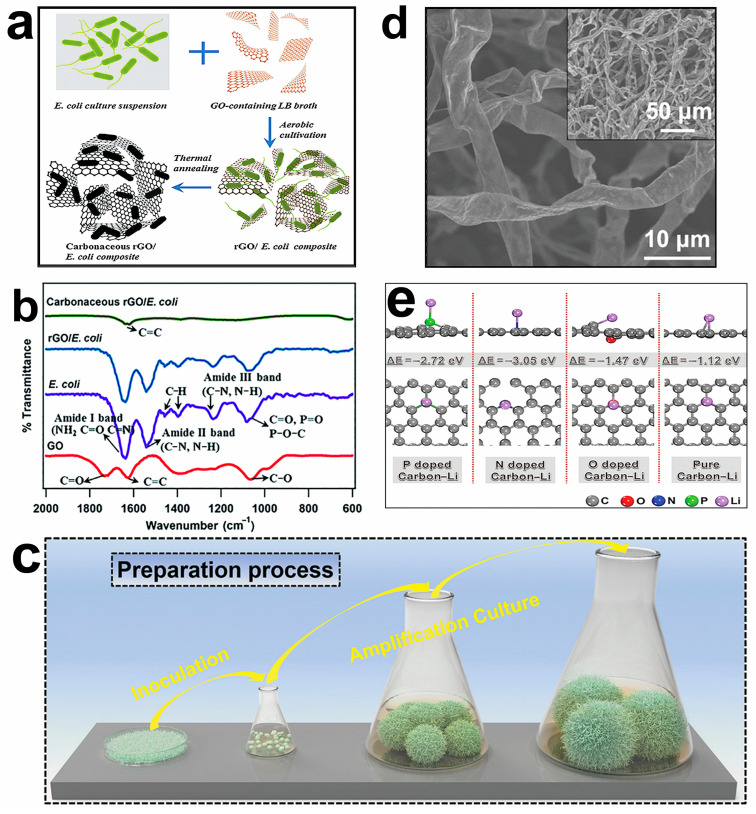
(**a**) Schematic showing the synthesis of carbonaceous rGO/*E. coli* composite. (**b**) Fourier-transform infrared spectroscopy spectra of the prepared samples. Reprinted with permission [126]. Copyright 2016, Royal Society of Chemistry. (**c**) Schematic fabrication procedure of *Rhizopus hyphae* balls; (**d**) scanning electron microscope image of *Rhizopus hyphae* carbon fiber from *Rhizopus hyphae*; (**e**) binding energies of Li atom with O-doped carbon, N-doped carbon, and P-doped carbon. Reprinted with permission [125]. Copyright 2022, Wiley.

Lithium batteries are a promising power generation device with lithium metal or alloy electrode material and nonaqueous electrolyte [117,118]. Lithium batteries have a high energy density, strong durability, and low self-discharge rate. Lithium can be used in various electronic devices and electric vehicle power generation systems [119,120,121]. The theoretical electrical capacity of the traditional lithium battery is relatively small and is extremely challenging to meet the demand for a large current [122]. Generally, lithium battery capacity is related to the porous structure, specific surface area, adsorption strength, and thermal conductivity of the electrode material [123]. Microorganisms, with the advantages of adsorption characteristics and rich nonmetallic elements, act as reductant or dopant sources, which can strongly interact with other components of the battery, enhancing their adsorption strength [124,125]. Microorganisms can be heteroatom sources doped into carbon nanomaterials. For instance, the porous carbonaceous rGO/*E. coli* composite material is prepared with doped N and P elements on graphene by aerobic culture and heat treatment (Figure 6a) [126]. The structure of this constructed electrode material presents a porous network with a large surface area, which attaches or encapsulates many *E. coli* cells on the rGO sheets and favors the recovery of the C=C bond, contributing to the high capacity and stability of lithium batteries (Figure 6b).

In addition, microorganisms can self-assemble electrode materials by their metabolism and rich surface functional groups [127]. In general, the growth mechanisms, size, composition, and electrical conductivity are essential factors affecting electrode properties [128]. Controlling the growth time of microorganisms and optimizing the composition of culture nutrient solution are effective ways to design efficient electrodes. Rhizome filaments can mutually self-assemble to form macroscopic 3D structures through spontaneous metabolic processes. Root filaments have many functional groups and can be self-assembled from bottom to top (Figure 6c). Regulating the culture conditions and carbonizations of root filaments can form multifunctional hollow carbon fiber. This centimeter-level porous carbon sphere has an interconnected porous structure, large surface area, and high electrical conductivity (Figure 6d). After codoping carbon fiber with N and P, the strong interaction with Li, S, and other atoms can enhance the chemosorption capacity of polysulfide, reduce the binding energy with lithium atoms, and accelerate the catalytic transformation of lithium–sulfur batteries (Figure 6e) [125].

### 4.4. Electroactive Microorganisms in MFCs

The traditional fuel cell involves the anode’s catalytic oxidation reaction and the cathode’s chemical reduction reaction [129,130]. Different from conventional fuel cells, the oxidation reaction of the MFC anode is the respiratory metabolism of microbial cells. It can transfer electrons with the external anode and directly convert chemical energy in the microbial body into electrical energy, which is significant in power generation and wastewater treatment [131]. The different types of electroactive microorganisms in the MFC system can completely oxidize various fuels with high energy efficiency and stability. MFCs also play a significant role in biological hydrogen production and biological remediation [132,133]. In general, the microorganisms located on the surface of the MFCs anode can form thicker biofilms and the electrons can be transferred from individual cells to the external electrodes, which can contribute to the MFC’s current and power. The biofilm anode is low-cost, environment-friendly, and easy to operate. The MFCs developed have limited power density, which can be ascribed to the low electron transfer efficiency on the anodic biofilm and the low loading capacity of the microorganisms [134,135]. In general, the extracellular electron transfer process of microorganisms involves electron transfer in multiple redox cycles, which severely limits the efficiency of the electron transfer and the power generation of MFCs. To break through the current power limitation of MFCs, regulating the density, thickness, electrical conductivity, biocompatibility, and microbial extracellular electron transfer of the anode electrode are effective ways to improve the power density and stability of MFCs.

**Figure 7:**
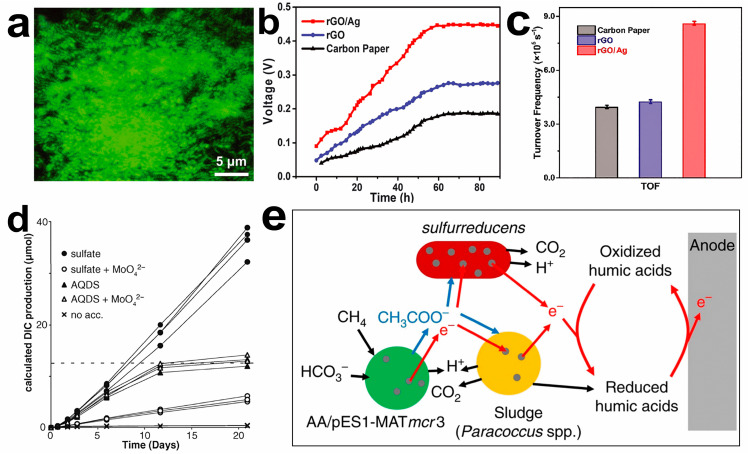
(**a**) Confocal laser scanning microscopy images of the *Shewanella* biofilms on rGO/Ag. (**b**) Voltage output of double-chamber MFCs with different electrodes. (**c**) Comparison of the calculated turnover frequencies for the biofilms on the carbon paper, rGO, and rGO/Ag electrodes. Reprinted with permission [137]. Copyright 2021, American Association for the Advancement of Science. (**d**) Digital image of production per vial in incubations with 1.0 cm^3^ of methane seep sediment. Reprinted with permission [138]. Copyright 2016 American Association for the Advancement of Science. (**e**) Proposed model of biological production of electricity from methane in MFCs. Reprinted with permission [139]. Copyright 2017, Springer Nature.

The mesoporous 3D structure with a high specific area and the doping of metal nanoparticles can be used to improve the electron transfer efficiency and bacterial loading of the cell electrodes. Carbon nanomaterials and their composites have a larger surface area and more gaps so that the electron transfer resistance can be effectively reduced and improve the bioelectricity generation by MFCs [136]. The embedded metal nanoparticles in microbial cells can promote the electron transfer process in the battery to enhance MFC current output [131]. A *Shewanella* MFC is proposed as a three-dimensional scaffold for reduced graphene oxide–silver nanoparticle (rGO/Ag) [137]. The rGO/Ag electrode can load many living cells and form highly compact biofilms on the anode surface, improving the electron extraction efficiency of *Shewanella* MFCs (Figure 7a). The output voltage of the two-compartment MFC based on rGO/Ag anode material is the highest and can reach a stable value within 48 h (Figure 7b). Based on the number of bacteria and the maximum current output of each electrode, the turnover frequency of rGO/Ag electrodes is significantly higher than other materials, which indicates that rGO/Ag electrodes have excellent electron extraction and transfer efficiency (Figure 7c). The rGO/Ag electrode can release Ag ions with positive charge, and microorganisms can reduce Ag ions into Ag nanoparticles, which help microorganisms transfer electrons to the anode, obtaining better power output and coulombic efficiency.

In addition, MFCs have attracted wide attention by using natural substances as raw materials for power generation. MFCs can be applied to convert methane into electricity directly; thus, biofuel is an efficient industrial upgrading strategy. Anaerobic methanotrophic bacteria can consume methane through metabolism, which is an important microbial metabolic process in the global carbon cycle. In the natural environment, anaerobic methanotrophic bacteria need symbiosis and cocultivation with sulfate-reducing bacteria. A strategy based on the growth of anaerobic methanotrophic bacteria alone is proposed (Figure 7d) [138]. This discovery provides a tremendous theoretical basis for power generation by methane MFCs. The deoxyribonucleic acid structure of anaerobic methanotrophic bacteria can be imitated and synthesized in the laboratory. Methane can be directly converted into electricity by indirect microbial contact on the anode, achieving 90% coulombic efficiency (Figure 7e) [139].

Recently, many researchers have found that a series of novel low-cost biosolar cells can be developed based on the characteristics of microorganisms, which can directly convert light into energy and obtain efficient current density [140,141]. Marine microbial ecosystems can be viewed as solar bioconversion systems. During the charging phase, photosynthetic microorganisms use solar energy to convert CO_2_ into organic matter. The energy in the organic matter is converted to electrical energy in the subsequent discharge phase. The researchers engineered a microbiome composed of cyanobacteria, *E. coli*, *Shewanella*, and *Geobacter*. The basic structure of the marine microbial ecosystem was simulated by genetic modification of the microbiome to form a small bionic ocean-battery with a high power of 380 μW and stable current output [142].

Overall, MFCs have been widely used in power generation, and Table 2 summarizes the recently reported MFCs. However, there are still imperfections in its structure and materials need to be improved [133].

## 5. Outlook and Perspective

Currently, the design of advanced energy technology with high efficiency and low cost has aroused extensive attention. Preparing traditional energy materials involves complex technological conditions and high preparation cost, which dramatically limits the broad application of energy materials in the industry. In this manuscript, based on the electron transfer mechanisms, redox, metabolism, and adsorption, and the rich structure and elemental composition of microorganisms, the relationship between microorganisms and electrode materials is established, and the application of electroactive microorganisms in energy materials (electrocatalyst, biological sensor, power generation device) and the current research progress are reviewed. Microbial corrosion, elemental doping, biological template, and biological self-assembly are adopted to construct superior electrode materials. The important factors of microorganisms toward advanced energy materials are analyzed from the biological characteristic and electrochemical performance relationship point of view. There are still some challenges to preparing advanced energy materials based on electroactive microorganisms.

First, the culture of electroactive microorganisms is limited by temperature and pH in the environment. The acquisition of specific microbial species requires strict separation and purification technologies. It is important to isolate specific microorganisms from a mixed natural microbial community and prevent contamination from other microorganisms. The selection of microbial species, the microbe–electrode compatibility, and the controllability of the preparation technology are the important conditions for constructing highly efficient energy devices. Electroactive microorganisms with simple culture conditions and easy activation can simplify the process and reduce the production cost. The heteroatomic materials with high content, such as nitrogen, phosphorus, and sulfur, show good electrochemical properties in energy storage and conversion devices. Moreover, selecting microorganisms abundant in amino acids and functional groups can provide nonmetallic elements, enrich the existing catalyst library, and provide innovative ideas for the modern development of energy technology and power generation technology.

Second, extracellular electron transfer between microorganisms and the electrode builds the bridge between characteristics of microorganisms and electrochemical catalysis. The precise electron transport mechanisms on the electrode biofilm are yet to be illustrated. Direct contact with higher energy has limited response sites to carry high output current. Various modification strategies have been studied to improve the disadvantages of low energy density, short lifetime, and instability in biotechnological applications. Nevertheless, the development of genetic engineering and synthetic biology techniques has limited indirect contact. Therefore, combining interdisciplinary fields, such as genomics, gene editing technology, cellular rebreathing, and nanotechnology, can regulate the extracellular electron transfer pipeline, improve biological metabolism, and secrete electron shuttle, promoting microbial biofilm formation and microbial intracellular metabolism. Relying on the natural ability of microorganisms to promote the electron transport process is also a promising method to enhance biotechnological performance.

Finally, advanced observation technology to analyze the structure–activity relationship of electroactive microbial materials is urgently demanded in practical application. In this regard, advanced in situ characterization techniques, such as in situ X-ray absorption, in situ Raman and Fourier transform infrared, etc., enable a comprehensive analysis of the transfer mechanism of microbial reaction intermediates and active sites. A theoretical calculation can also be adopted to analyze the electronic structure of multiple electron systems at the atomic scale. This would enable exploration of the electrochemical properties of energy materials and the optimal adsorption/desorption energy for the reaction process from the structure–activity relationship.

## Figures and Tables

**Table 1:** The analytes and sensitivities of different types of microbial sensors.

Bacteria	Target Analyte	Sensitivity	Ref.
*E. coli*	Arsenic	0.74–69 µg L^−1^	[86]
*E. coli*	Arsentate	<10 µg L^−1^	[87]
*E. coli*	Benzene, toluene, and xylene	40 µM	[88]
*E. coli*	Chromate	100 nM	[89]
*Deinococcus radiodurans*	Cadmium	1–10 mM	[90]
*Salmonella typhimurium*	Single-stranded DNA	10 nM mitomycinC	[91]
*Pseudomonas putida*	Phenol	3 µM	[92]
*Burkholderia sartisoli*	Naphthalene and phenanthrene	0.17 µM	[93]
*Saccharomyces cerevisiae*	Nitrosamine	>1 × 10^−10^ g L^−1^	[94]
*Agrobacterium*	2,4-Dichlorophenoxyacetic	12.5 µM	[95]

**Table 2:** The electrochemical properties of MFCs with different electrode materials and bacteria species.

Electrode	Bacteria	Culture	Current Density (mA cm^−2^)	Power Density (mW cm^−2^)	QE	Ref.
Graphite fiber brush	Mix	/	0.8	0.143	/	[143]
Carbon paper	Mix	/	0.28	0.05	/	[143]
Graphene/PANI	*Shewanella* MR-1	Luria–Bertani broth medium	0.58	0.031	/	[144]
VA-CNT	*Geobacter*	Sodium acetate medium	0.26	0.083	61%	[145]
CNT textile fiber	Mix	Sodium acetate medium (conductivity: 7.0 mS cm^−1^)	0.5	0.11	/	[146]
rGO/Pt	*Shewanella* MR-1	M9 buffer solution containing 18 mM sodium (fed-batch mode)	0.69	0.148	69%	[147]
3D chitosan hydrogel	*Pseudomonas* *aeruginosa*	Luria–Bertani broth medium	0.55	0.153	/	[148]
rGO/Ag	*Shewanella* MR-1	The medium with lactate as the nutrient (fed-batch mode)	3.85	0.66	81%	[137]
Steel/CNT	*Geobacter*	Sodium acetate medium	/	/	16%	[149]
Carbon fiber/Ti	Mix	Nitrate-containing MBG11-S medium (genetic engineering)	0.73	0.52	/	[142]

## Data Availability

Not applicable.

## References

[1] Shi F.Y., Zhai L.L., Liu Q.Q., Yu J.Y., Lau S.P., Xia B.Y., Xu Z.L. (2023). Emerging catalytic materials for practical lithium-sulfur batteries. J. Energy Chem..

[2] Shen T., Wang S., Zhao T., Hu Y., Wang D. (2022). Recent advances of single-atom-alloy for energy electrocatalysis. Adv. Energy Mater..

[3] Yang H., Dong C.L., Wang H.M., Qi R.J., Gong L.Q., Lu Y.R., He C.H., Chen S.H., You B., Liu H.F. (2022). Constructing nickel-iron oxyhydroxides integrated with iron oxides by microorganism corrosion for oxygen evolution. Proc. Natl. Acad. Sci. USA.

[4] Wang Z.Y., Wu Y.L., Xu K.Q., Jiang L.P., Sun J.K., Cai G.Y., Li G.F., Xia B.Y., Liu H.F. (2021). Hierarchical oriented metal-organic frameworks assemblies for water-evaporation induced electricity generation. Adv. Funct. Mater..

[5] Chen C., Tian H., Fu Z.Q., Cui X.Z., Kong F.T., Meng G., Chen Y.F., Qi F.G., Chang Z.W., Zhu L.B. (2022). Pt NPs-loaded siloxene nanosheets for hydrogen co-evolutions from Zn-H_2_O fuel cells-powered water-splitting. Appl. Catal. B—Environ..

[6] Luo H., Barrio J., Sunny N., Li A., Steier L., Shah N., Stephens I.E.L., Titirici M.M. (2021). Progress and perspectives in photo-and electrochemical-oxidation of biomass for sustainable chemicals and hydrogen production. Adv. Energy Mater..

[7] Yang H., Hu H.L., Xia B.Y. (2022). Progress on nanostructured gel catalysts for oxygen electrocatalysis. Nano Res..

[8] Hu H.L., Chen Y.N., You F., Yao J.L., Yang H., Jiang X.L., Xia B.Y. (2023). Recycling and upgrading utilization of polymer plastics. Chin. J. Chem..

[9] Fu G.T., Xia B.Y., Ma R.G., Chen Y., Tang Y.W., Lee J.M. (2015). Trimetallic PtAgCu@PtCu core@shell concave nanooctahedrons with enhanced activity for formic acid oxidation reaction. Nano Energy.

[10] Zhang J.Y., He T., Wang M.D., Qi R.J., Yan Y., Dong Z.H., Liu H.F., Wang H.M., Xia B.Y. (2019). Energy-saving hydrogen production coupling urea oxidation over a bifunctional nickel-molybdenum nanotube array. Nano Energy.

[11] Xie S., Sun B.W., Sun H., Zhan K., Zhao B., Yan Y., Xia B.Y. (2019). Engineering of molybdenum sulfide nanostructures towards efficient electrocatalytic hydrogen evolution. Int. J. Hydrogen Energ..

[12] Li Z., Yan Y., Xu S.M., Zhou H., Xu M., Ma L., Shao M., Kong X., Wang B., Zheng L. (2022). Alcohols electrooxidation coupled with H_2_ production at high current densities promoted by a cooperative catalyst. Nat. Commun..

[13] Abi A., Mohammadpour Z., Zuo X., Safavi A. (2018). Nucleic acid-based electrochemical nanobiosensors. Biosens. Bioelectron..

[14] Jiang X.L., Chen Y.N., Zhang X., You F., Yao J.L., Yang H., Xia B.Y. (2022). Magnetic field-assisted construction and enhancement of electrocatalysts. Chem. Sus. Chem..

[15] Yang X.X., Deng P.L., Liu D.Y., Zhao S., Li D., Wu H., Ma Y.M., Xia B.Y., Li M.T., Xiao C.H. (2020). Partial sulfuration-induced defect and interface tailoring on bismuth oxide for promoting electrocatalytic CO_2_ reduction. J. Mater. Chem. A.

[16] Santovito E., Elisseeva S., Smyth C., Cruz-Romero M., Kerry J.P., Duffy G., Papkovsky D.B. (2022). A sensor-based system for rapid on-site testing of microbial contamination in meat samples and carcasses. J. Appl. Microbiol..

[17] Shi Z., Wu X., Zou Z., Yu L., Hu F., Li Y., Guo C., Li C.M. (2021). Screen-printed analytical strip constructed with bacteria-templated porous N-doped carbon nanorods/Au nanoparticles for sensitive electrochemical detection of dopamine molecules. Biosens. Bioelectron..

[18] Ozden S., Macwan I.G., Owuor P.S., Kosolwattana S., Autreto P.A.S., Silwal S., Vajtai R., Tiwary C.S., Patra P.K., Ajayan P.M. (2017). Bacteria as bio-template for 3D carbon nanotube architectures. Sci. Rep..

[19] Wei X., Li Y., Chen L., Shi J. (2021). Formic acid electro-synthesis by concurrent cathodic CO_2_ reduction and anodic CH_3_OH oxidation. Angew. Chem. Int. Ed..

[20] Yu J., Yu W., Chang B., Li X., Jia J., Wang D., Xu Z., Zhang X., Liu H., Zhou W. (2022). Waste-yeast biomass as nitrogen/phosphorus sources and carbon template: Environment-friendly synthesis of N, P-Mo_2_C nanoparticles on porous carbon matrix for efficient hydrogen evolution. Chin. Chem. Lett..

[21] Liu J., Zheng Y., Hong Z., Cai K., Zhao F., Han H. (2016). Microbial synthesis of highly dispersed PdAu alloy for enhanced electrocatalysis. Sci. Adv..

[22] Zhang S., Zhou H., Liao H., Tan P., Tian W., Pan J. (2022). Microbial synthesis of efficient palladium electrocatalyst with high loadings for oxygen reduction reaction in acidic medium. J. Colloid Interf. Sci..

[23] Yu J., Chang B., Yu W., Li X., Wang D., Xu Z., Zhang X., Liu H., Zhou W. (2022). Chromium phosphide nanoparticles embedded in porous nitrogen-/phosphorus-doped carbon as efficient electrocatalysts for a nitrogen reduction reaction. Carbon Energy.

[24] Cao J., Sun Z., Li J., Zhu Y., Yuan Z., Zhang Y., Li D., Wang L., Han W. (2021). Microbe-assisted assembly of Ti_3_C_2_T_x_ MXene on fungi-derived nanoribbon heterostructures for ultrastable sodium and potassium ion storage. ACS Nano.

[25] Dong J., Fernández-Fueyo E., Hollmann F., Paul C.E., Pesic M., Schmidt S., Wang Y., Younes S., Zhang W. (2018). Biocatalytic oxidation reactions: A chemist’s perspective. Angew. Chem. Int. Ed..

[26] Evans R.M., Siritanaratkul B., Megarity C.F., Pandey K., Esterle T.F., Badiani S., Armstrong F.A. (2019). The value of enzymes in solar fuels research–efficient electrocatalysts through evolution. Chem. Soc. Rev..

[27] Zhang N., Müller B., Kirkeby T.Ø., Kara S., Loderer C. (2022). Development of a thioredoxin-based cofactor regeneration system for NADPH-dependent oxidoreductases. Chem. Cat. Chem..

[28] Feng X., Qin S., Zhang D., Chen P., Hu J., Wang G., Liu Y., Wei B., Li Q., Yang Y. (2022). Nitrogen input enhances microbial carbon use efficiency by altering plant-microbe-mineral interactions. Glob. Chang. Biol..

[29] He K., Li W., Tang L., Li W., Lv S., Xing D. (2022). Suppressing methane production to boost high-purity hydrogen production in microbial electrolysis cells. Environ. Sci. Technol..

[30] Yao H., Wang X., Li K., Li C., Zhang C., Zhou J., Cao Z., Wang H., Gu M., Huang M. (2022). Strong electronic coupling between ruthenium single atoms and ultrafine nanoclusters enables economical and effective hydrogen production. Appl. Catal. B.

[31] Lee S.Y., Kim H.U., Chae T.U., Cho J.S., Kim J.W., Shin J.H., Kim D.I., Ko Y.S., Jang W.D., Jang Y.S. (2019). A comprehensive metabolic map for production of bio-based chemicals. Nat. Catal..

[32] Montaño López J., Duran L., Avalos J.L. (2022). Physiological limitations and opportunities in microbial metabolic engineering. Nat. Rev. Microbiol..

[33] Wang B., Bonné R., Zhang Y., Wang A., Liu W. (2022). Renewable energy driving microbial electrochemistry toward carbon neutral. Sustain. Horiz..

[34] Ter Heijne A. (2020). Bioelectrochemistry for flexible control of biological processes. Environ. Sci. Ecotechnol..

[35] Zhang D., Fan B., Ying L., Li N., Brabec C.J., Huang F., Cao Y. (2021). Recent progress in thick-film organic photovoltaic devices: Materials, devices, and processing. Sus. Mat..

[36] Liu X., Zhang K., Sun Y. (2023). Upgrading CO_2_ into acetate on Bi_2_O_3_@carbon felt integrated electrode via coupling electrocatalysis with microbial synthesis. Sus. Mat..

[37] Roy S., Pandit S., Mohan S.V., Varjani S., Pandey A. (2019). 1.2-Microbial electrochemical system: Principles and application. Microbial Electrochemical Technology.

[38] Zhang L., Yuan S.M., Yang L.M., Fang Z., Zhao G.C. (2013). An enzymatic glucose biosensor based on a glassy carbon electrode modified with manganese dioxide nanowires. Microchim. Acta.

[39] Mitrova B., Waffo A.F.T., Kaufmann P., Iobbi-Nivol C., Leimkühler S., Wollenberger U. (2018). Trimethylamine N-oxide electrochemical biosensor with a Chimeric Enzyme. Chem. Electro. Chem..

[40] Chen H., Simoska O., Lim K., Grattieri M., Yuan M., Dong F., Lee Y.S., Beaver K., Weliwatte S., Gaffney E.M. (2020). Fundamentals, applications, and future directions of bioelectrocatalysis. Chem. Rev..

[41] Zou L., Qiao Y., Li C.M. (2018). Boosting microbial electrocatalytic kinetics for high power fensity: Insights into synthetic biology and advanced nanoscience. Electrochem. Energy R..

[42] Wang Z., Liu S., Wu P., Cai C.J.A.C. (2009). Detection of glucose based on direct electron transfer reaction of glucose oxidase immobilized on highly ordered polyaniline nanotubes. Anal. Chem..

[43] Jung J.H., Cheon D.S., Liu F., Lee K.B., Seo T.S. (2010). A graphene oxide based immuno-biosensor for pathogen detection. Angew. Chem. Int. Ed..

[44] Ma J., Du M., Wang C., Xie X., Wang H., Zhang Q. (2021). Advances in airborne microorganisms detection using biosensors: A critical review. Front. Environ. Sci. Eng..

[45] Klein R.D., Hultgren S.J. (2020). Urinary tract infections: Microbial pathogenesis, host-pathogen interactions and new treatment strategies. Nat. Rev. Microbiol..

[46] Yao Y., Xie G., Zhang X., Yuan J., Hou Y., Chen H. (2021). Fast detection of *E. coli* with a novel fluorescent biosensor based on a FRET system between UCNPs and GO@Fe_3_O_4_ in urine specimens. Anal. Methods.

[47] Logan B.E., Rossi R., Ragab A., Saikaly P.E. (2019). Electroactive microorganisms in bioelectrochemical systems. Nat. Rev. Microbiol..

[48] Wen A.M., Steinmetz N.F. (2016). Design of virus-based nanomaterials for medicine, biotechnology, and energy. Chem. Soc. Rev..

[49] Xu Y.N., Cui X.S., Tae J.C., Jin Y.X., Kim N.H. (2011). DNA synthesis and epigenetic modification during mouse oocyte fertilization by human or hamster sperm injection. J. Assist. Reprod. Genet..

[50] Hu J., Yuan X., Wang C., Shao X., Yang B., Abdul Razzaq A., Zhao X., Lian Y., Deng Z., Chen M. (2020). Self-phosphorization of MOF-armored microbes for advanced energy storage. Small.

[51] Wang X., Pu J., Liu Y., Ba F., Cui M., Li K., Xie Y., Nie Y., Mi Q., Li T. (2019). Immobilization of functional nano-objects in living engineered bacterial biofilms for catalytic applications. Nat. Sci. Rev..

[52] Kalathil S., Katuri K.P., Alazmi A.S., Pedireddy S., Kornienko N., Costa P.M.F.J., Saikaly P.E. (2019). Bioinspired synthesis of reduced graphene oxide-wrapped *Geobacter sulfurreducens* as a hybrid electrocatalyst for efficient oxygen evolution reaction. Chem. Mater..

[53] Hou G., Jia X., Kang H., Qiao X., Liu Y., Li Y., Wu X., Qin W. (2022). CoNi nano-alloys modified yolk-shell structure carbon cage via Saccharomycetes as carbon template for efficient oxygen evolution reaction. Appl. Catal. B.

[54] Pankratova G., Hederstedt L., Gorton L. (2019). Extracellular electron transfer features of Gram-positive bacteria. Analytica Chimica Acta..

[55] Thapa B.S., Kim T., Pandit S., Song Y.E., Afsharian Y.P., Rahimnejad M., Kim J.R., Oh S.E. (2022). Overview of electroactive microorganisms and electron transfer mechanisms in microbial electrochemistry. Bioresour. Technol..

[56] Liu X., Gao H., Ward J.E., Liu X., Yin B., Fu T., Chen J., Lovley D.R., Yao J. (2020). Power generation from ambient humidity using protein nanowires. Nature.

[57] Myers C.R., Nealson K.H. (1988). Microbial reduction of manganese oxides: Interactions with iron and sulfur. Geochim. Cosmochim. Ac..

[58] Lovley D.R., Phillips E.J. (1988). Novel mode of microbial energy metabolism: Organic carbon oxidation coupled to dissimilatory reduction of iron or manganese. Appl. Environ. Microbiol..

[59] Vaidyanathan H., Kandasamy V., Gopal Ramakrishnan G., Ramachandran K.B., Jayaraman G., Ramalingam S. (2011). Glycerol conversion to 1, 3-Propanediol is enhanced by the expression of a heterologous alcohol dehydrogenase gene in *Lactobacillus reuteri*. AMB Express.

[60] Zhao J., Li F., Cao Y., Zhang X., Chen T., Song H., Wang Z. (2021). Microbial extracellular electron transfer and strategies for engineering electroactive microorganisms. Biotechnol. Adv..

[61] Çelekli A., Bozkurt H. (2011). Bio-sorption of cadmium and nickel ions using *Spirulina platensis*: Kinetic and equilibrium studies. Desalination.

[62] Yang C., Zhu Y., Liu J., Qin Y., Hu W.J.N.E. (2020). Defect engineering for electrochemical nitrogen reduction reaction to ammonia. Nano Energy.

[63] Wang N., Li X., Hu M.K., Wei W., Zhou S.H., Wu X.T., Zhu Q.L. (2022). Ordered macroporous superstructure of bifunctional cobalt phosphide with heteroatomic modification for paired hydrogen production and polyethylene terephthalate plastic recycling. Appl. Catal. B.

[64] Li G., Wang J., Yu J., Liu H., Cao Q., Du J., Zhao L., Jia J., Liu H., Zhou W. (2020). Ni-Ni_3_P nanoparticles embedded into N, P-doped carbon on 3D graphene frameworks via in situ phosphatization of saccharomycetes with multifunctional electrodes for electrocatalytic hydrogen production and anodic degradation. Appl. Catal. B.

[65] Guo X., Qian C., Wan X., Zhang W., Zhu H., Zhang J., Yang H., Lin S., Kong Q., Fan T. (2020). Facile in situ fabrication of biomorphic Co_2_P-Co_3_O_4_/rGO/C as an efficient electrocatalyst for the oxygen reduction reaction. Nanoscale.

[66] Zhou W., Xiong T., Shi C., Zhou J., Zhou K., Zhu N., Li L., Tang Z., Chen S. (2016). Bioreduction of precious metals by microorganism: Efficient gold@N-doped carbon electrocatalysts for the hydrogen evolution reaction. Angew. Chem. Int. Ed. Engl..

[67] Yu J., Li G., Liu H., Zhao L., Wang A., Liu Z., Li H., Liu H., Hu Y., Zhou W. (2019). Ru–Ru_2_PΦNPC and NPC@RuO_2_ synthesized via environment-friendly and solid-phase phosphating process by saccharomycetes as N/P Sources and carbon template for overall water splitting in acid electrolyte. Adv. Funct. Mater..

[68] Lovley D.R., Holmes D.E. (2022). Electromicrobiology: The ecophysiology of phylogenetically diverse electroactive microorganisms. Nat. Rev. Microbiol..

[69] Shi C., Gu W., Zhu N. (2020). Intensification of sorption–reduction coupled gold biorecovery process through microbial surface modification: Effect on gold sorption and reduction. World J. Microb. Biot..

[70] Song Y., Lee J., Shin J., Cho B. (2020). Functional cooperation of the glycine synthase-reductase and Wood–Ljungdahl pathways for autotrophic growth of *Clostridium drakei*. Proc. Natl. Acad. Sci. USA.

[71] Kim J., Cestellos-Blanco S., Shen Y.X., Cai R., Yang P. (2022). Enhancing biohybrid CO_2_ to multicarbon reduction via adapted whole-cell catalysts. Nano Lett..

[72] Lovley D.R. (2008). The microbe electric: Conversion of organic matter to electricity. Curr. Opin. Biotechnol..

[73] Zhang G., Liang D., Zhao Z., Qi J., Huang L. (2022). Enhanced performance of microbial fuel cell with electron mediators from tetracycline hydrochloride degradation. Environ. Res..

[74] Chen Y., Zhang B., Wu D., Li F., Song H. (2020). Research progress in screening method of exoelectrogens. J. Biotechnol..

[75] Ni G., Simone D., Palma D., Broman E., Wu X., Turner S., Dopson M. (2018). A novel inorganic sulfur compound metabolizing ferroplasma-like population is suggested to mediate extracellular electron transfer. Front. Microbiol..

[76] Saunders S.H., Tse E.C.M., Yates M.D., Otero F.J., Trammell S.A., Stemp E.D.A., Barton J.K., Tender L.M., Newman D.K. (2020). Extracellular DNA promotes efficient extracellular electron transfer by pyocyanin in pseudomonas aeruginosa biofilms. Cell.

[77] Li S., Zhu L., Lin S., Xu W. (2023). Toehold-mediated biosensors: Types, mechanisms and biosensing strategies. Biosens. Bioelectron..

[78] Kumar R. (2020). NiCo_2_O_4_ nano-/microstructures as high-performance biosensors: A review. Nano-Micro Lett..

[79] Yang H., Jia X., Han Y. (2022). Microbial redox coenzyme engineering and applications in biosynthesis. Trends Microbiol..

[80] Zachos I., Döring M., Tafertshofer G., Simon R.C., Sieber V. (2021). Carba nicotinamide adenine dinucleotide phosphate: Robust cofactor for redox biocatalysis. Angew. Chem. Int. Edit..

[81] Dai Y., Liu C.C. (2019). Recent advances on electrochemical biosensing strategies toward universal point-of-care systems. Angew. Chem. Int. Edit..

[82] Monser L., Adhoum N.J.S., Technology P. (2002). Modified activated carbon for the removal of copper, zinc, chromium and cyanide from wastewater. Sep. Purif. Technol..

[83] Kim M., Lim J.W., Kim H.J., Lee S.K., Lee S.J., Kim T. (2015). Chemostat-like microfluidic platform for highly sensitive detection of heavy metal ions using microbial biosensors. Biosens. Bioelectron..

[84] Turner P.F. (2013). Biosensors: Sense and sensibility. Chem. Soc. Rev..

[85] Xu X., Ying Y. (2011). Microbial biosensors for environmental monitoring and food analysis. Food Rev. Int..

[86] Sharma P., Asad S., Ali A. (2013). Bioluminescent bioreporter for assessment of arsenic contamination in water samples of India. J. Biosci..

[87] de Mora K., Joshi N., Balint B.L. (2011). A pH-based biosensor for detection of arsenic in drinking water. Anal. Bioanal. Chem..

[88] Willardson B., Wilkins J., Rand T., Schupp J., Hill K., Keim P., Jackson P. (1998). Development and testing of a bacterial biosensor for toluene-based environmental contaminants. Appl. Environ. Microb..

[89] Branco R., Cristóvão A., Morais P.V. (2013). Highly sensitive, highly specific whole-cell bioreporters for the detection of chromate in environmental samples. PLoS ONE.

[90] Joe M., Lee K., Lim S., Im S., Song H., Lee I., Kim D. (2012). Pigment-based whole-cell biosensor system for cadmium detection using genetically engineered *Deinococcus radiodurans*. Bioproc. Biosyst. Eng..

[91] Nakamura S., Oda Y., Shimada T., Oki I., Sugimoto K. (1987). SOS-inducing activity of chemical carcinogens and mutagens in *Salmonella typhimurium* TA1535/pSK1002: Examination with 151 chemicals. Mutat. Res. Lett..

[92] Shingler V., Moore T. (1994). Sensing of aromatic compounds by the DmpR transcriptional activator of phenol-catabolizing *Pseudomonas* sp. strain CF600. J. Bacteriol..

[93] Tecon R., Beggah S., Czechowska K., Sentchilo V., Chronopoulou P., McGenity T. (2010). Development of a multistrain bacterial bioreporter platform for the monitoring of hydrocarbon contaminants in marine environments. Environ. Sci. Technol..

[94] Wang H.C., Wang Y.X., Ding H.T., Li J., Cheng C., Zheng F. (2022). Oral metal–organic gel protected whole-cell biosensor for in situ monitoring nitrosamines in the gastrointestinal Tract. Nano Lett..

[95] Ritcharoon B., Sallabhan R., Toewiwat N., Mongkolsuk S., Loprasert S. (2020). Detection of 2,4-dichlorophenoxyacetic acid herbicide using a FGE-sulfatase based whole-cell agrobacterium biosensor. J. Microbiol. Meth..

[96] Hassan R.A., Heng L.Y., Ahmad A., Tan L.L. (2019). Rapid determination of kappa-carrageenan using a biosensor from immobilized *Pseudomonas carrageenovora* cells. PLoS ONE.

[97] Gui Q., Lawson T., Shan S., Yan L., Liu Y. (2017). The application of whole cell-based biosensors for use in environmental analysis and in medical diagnostics. Sensors.

[98] Liu B., Lei Y., Li B. (2014). A batch-mode cube microbial fuel cell based "shock" biosensor for wastewater quality monitoring. Biosens. Bioelectron..

[99] Yin J., Zhu Y., Liang Y., Luo Y., Lou J., Hu X., Meng Q., Zhu T., Yu Z. (2022). Development of whole-cell biosensors for screening of peptidoglycan-targeting antibiotics in a Gram-negative bacterium. Appl. Environ. Microbiol..

[100] Wang H., Song D., Chen Y., Xu W., Han X., Zhu A., Long F. (2022). Development of portable whole-cell biosensing platform with lyophilized bacteria and its application for rapid on-site detection of heavy metal toxicity without pre-resuscitation. Anal. Chim. Acta.

[101] Liu C., Yu H., Zhang B., Liu S., Liu C.-g., Li F., Song H. (2022). Engineering whole-cell microbial biosensors: Design principles and applications in monitoring and treatment of heavy metals and organic pollutants. Biotechnol. Adv..

[102] Guo M., Du R., Xie Z., He X., Huang K., Luo Y., Xu W. (2019). Using the promoters of MerR family proteins as “rheostats” to engineer whole-cell heavy metal biosensors with adjustable sensitivity. J. Biol. Eng..

[103] Zhao Y., Huang J., Huang Q., Tao Y., Gu R., Li H.-Y., Liu H. (2022). Electrochemical biosensor employing PbS colloidal quantum dots/Au nanospheres-modified electrode for ultrasensitive glucose detection. Nano Res..

[104] Wan X., Volpetti F., Petrova E., French C., Maerkl S.J., Wang B. (2019). Cascaded amplifying circuits enable ultrasensitive cellular sensors for toxic metals. Nat. Chem. Biol..

[105] Xu Z., Liu B., Dong Q., Lei Y., Li Y., Ren J., McCutcheon J., Li B. (2015). Flat microliter membrane-based microbial fuel cell as “on-line sticker sensor” for self-supported in situ monitoring of wastewater shocks. Bioresour. Technol..

[106] Li M., Zhou M., Tian X., Tan C., McDaniel C.T., Hassett D.J., Gu T. (2018). Microbial fuel cell (MFC) power performance improvement through enhanced microbial electrogenicity. Biotechnol. Adv..

[107] Castellano-Hinojosa A., González-Martínez A., Pozo C., González-López J. (2022). Diversity of electroactive and non-electroactive microorganisms and their potential relationships in microbial electrochemical systems: A review. J. Water Process Eng..

[108] Ruff A., Conzuelo F., Schuhmann W. (2020). Bioelectrocatalysis as the basis for the design of enzyme-based biofuel cells and semi-artificial biophotoelectrodes. Nat. Catal..

[109] Lovley D.R. (2017). e-Biologics: Fabrication of sustainable electronics with “Green” biological materials. mBio.

[110] Paquete C.M., Rosenbaum M.A., Bañeras L., Rotaru A.E., Puig S. (2022). Let’s chat: Communication between electroactive microorganisms. Bioresour. Technol..

[111] Gahlawat G., Choudhury A.R. (2019). A review on the biosynthesis of metal and metal salt nanoparticles by microbes. RSC Adv..

[112] Shapiro D.M., Mandava G., Yalcin S.E., Arranz-Gibert P., Dahl P.J., Shipps C., Gu Y., Srikanth V., Salazar-Morales A.I., O’Brien J.P. (2022). Protein nanowires with tunable functionality and programmable self-assembly using sequence-controlled synthesis. Nat. Commun..

[113] Reguera G., McCarthy K.D., Mehta T., Nicoll J.S., Tuominen M.T., Lovley D.R. (2005). Extracellular electron transfer via microbial nanowires. Nature.

[114] Tan Y., Adhikari R.Y., Malvankar N.S., Pi S., Ward J.E., Woodard T.L., Nevin K.P., Xia Q., Tuominen M.T., Lovley D.R. (2016). Synthetic biological protein nanowires with high conductivity. Small.

[115] Xue G., Xu Y., Ding T., Li J., Yin J., Fei W., Cao Y., Yu J., Yuan L., Gong L. (2017). Water-evaporation-induced electricity with nanostructured carbon materials. Nat. Nanotechnol..

[116] Srivastava S.K., Piwek P., Ayakar S.R., Bonakdarpour A., Wilkinson D.P., Yadav V.G. (2018). A biogenic photovoltaic material. Small.

[117] Wang X.T., Gu Z.Y., Ang E.H., Zhao X.X., Wu X.L., Liu Y. (2022). Prospects for managing end-of-life lithium-ion batteries: Present and future. Interdiscip. Mater..

[118] Huang Y.J.I.M. (2022). The discovery of cathode materials for lithium-ion batteries from the view of interdisciplinarity. Interdiscip. Mater..

[119] Ai G., Dai Y., Mao W., Zhao H., Fu Y., Song X., En Y., Battaglia V.S., Srinivasan V., Liu G. (2016). Biomimetic ant-nest electrode structures for high sulfur ratio lithium–sulfur batteries. Nano Lett..

[120] Wei J., Zhou M., Long A., Xue Y., Liao H., Wei C., Xu Z.J. (2018). Heterostructured electrocatalysts for hydrogen evolution reaction under alkaline conditions. Nano-Micro Lett..

[121] Cao D., Zhang Q., Hafez A.M., Jiao Y., Ma Y., Li H., Cheng Z., Niu C., Zhu H. (2019). Lignin-derived holey, layered, and thermally conductive 3D scaffold for lithium dendrite suppression. Small Methods.

[122] Mikhaylik Y.V., Akridge J.R. (2004). Polysulfide shuttle study in the Li/S battery system. J. Electrochem. Soc..

[123] Liu N., Li W., Pasta M., Cui Y. (2014). Nanomaterials for electrochemical energy storage. Front. Phys..

[124] Wen Y., Wang B., Huang C., Wang L., Hulicova-Jurcakova D. (2015). Synthesis of phosphorus-doped graphene and its wide potential window in aqueous supercapacitors. Chemstry.

[125] Huang L., Shen S., Zhong Y., Zhang Y., Zhang L., Wang X., Xia X., Tong X., Zhou J., Tu J. (2022). Multifunctional hyphae carbon powering lithium–sulfur batteries. Adv. Mater..

[126] Wang X., Ai W., Li N., Yu T., Chen P. (2015). Graphene–bacteria composite for oxygen reduction and lithium ion batteries. J. Mater. Chem. A.

[127] Cheah L.C., Stark T., Adamson L.S.R., Abidin R.S., Lau Y.H., Sainsbury F., Vickers C.E. (2021). Artificial self-assembling nanocompartment for organizing metabolic pathways in yeast. ACS Synth. Biol..

[128] Yu W., Bai H., Zeng Y., Zhao H., Xia S., Huang Y., Lv F., Wang S. (2022). Solar-driven producing of value-added chemicals with organic semiconductor-bacteria biohybrid system. Research.

[129] Logan B.E. (2009). Exoelectrogenic bacteria that power microbial fuel cells. Nat. Rev. Microbiol..

[130] Dutta R.R., Puzari P. (2014). Amperometric biosensing of organophosphate and organocarbamate pesticides utilizing polypyrrole entrapped acetylcholinesterase electrode. Biosens. Bioelectron..

[131] Ke Z., Yuan X.S., Feng X.L., Guo S.P. (2022). Bimetallic catalysts as electrocatalytic cathode materials for the oxygen reduction reaction in microbial fuel cell: A review. Green Energy Environ..

[132] Song H.L., Zhang C., Lu Y.X., Li H., Shao Y., Yang Y.L. (2022). Enhanced removal of antibiotics and antibiotic resistance genes in a soil microbial fuel cell via in situ remediation of agricultural soils with multiple antibiotics. Sci. Total Environ..

[133] Shrivastava A., Sharma R.K. (2022). Lignocellulosic biomass based microbial fuel cells: Performance and applications. J. Clean. Prod..

[134] Chae K.J., Choi M.J., Lee J.W., Kim K.Y., Kim I.S. (2009). Effect of different substrates on the performance, bacterial diversity, and bacterial viability in microbial fuel cells. Bioresour. Technol..

[135] Lu N., Li L., Wang C., Wang Z., Wang Y., Yan Y., Qu J., Guan J. (2021). Simultaneous enhancement of power generation and chlorophenol degradation in nonmodified microbial fuel cells using an electroactive biofilm carbon felt anode. Sci. Total Environ..

[136] Peng L., Peng H., Li W., Zhao D. (2022). Monomicellar assembly to synthesize structured and functional mesoporous carbonaceous nanomaterials. Nat. Protoc..

[137] Cao B., Zhao Z., Peng L., Shiu H.Y., Ding M., Song F., Guan X., Lee C.K., Huang J., Zhu D. (2021). Silver nanoparticles boost charge-extraction efficiency in Shewanella microbial fuel cells. Science.

[138] Scheller S., Yu H., Chadwick G.L., McGlynn S.E., Orphan V.J. (2016). Artificial electron acceptors decouple archaeal methane oxidation from sulfate reduction. Science.

[139] McAnulty M.J., Poosarla V.G., Kim K.Y., Jasso-Chavez R., Logan B.E., Wood T.K. (2017). Electricity from methane by reversing methanogenesis. Nat. Commun..

[140] Mohammadifar M., Tahernia M., Choi S. (2020). A miniaturized, self-sustaining, and integrable bio-solar power system. Nano Energy.

[141] Liu L., Choi S. (2017). Self-sustaining, solar-driven bioelectricity generation in micro-sized microbial fuel cell using co-culture of heterotrophic and photosynthetic bacteria. J. Power Sources.

[142] Zhu H., Xu L., Luan G. (2022). A miniaturized bionic ocean-battery mimicking the structure of marine microbial ecosystems. Nat. Commun..

[143] Liu H., Ramnarayanan R., Logan B.E. (2004). Production of electricity during wastewater treatment using a single chamber microbial fuel cell. Environ. Sci. Technol..

[144] Zhao C., Gai P., Liu C. (2013). Polyaniline networks grown on graphene nanoribbons-coated carbon paper with a synergistic effect for high-performance microbial fuel cells. J. Mater. Chem. A.

[145] Ren H., Pyo S., Lee J.I., Park T.J., Gittleson F.S., Leung F.C., Kim J., Taylor A.D., Lee H.S., Chae J. (2015). A high power density miniaturized microbial fuel cell having carbon nanotube anodes. J. Power Sources.

[146] Yuan Y., Zhou S., Liu Y., Tang J. (2013). Nanostructured macroporous bioanode based on polyaniline-modified natural loofah sponge for high-performance microbial fuel cells. Environ. Sci. Technol..

[147] Zhao S., Li Y., Yin H. (2015). Three-dimensional graphene/Pt nanoparticle composites as freestanding anode for enhancing performance of microbial fuel cells. Sci. Adv..

[148] He Z., Liu J., Qiao Y., Li C.M., Tan T.T.Y. (2012). Architecture engineering of hierarchically porous chitosan/vacuum-stripped graphene scaffold as bioanode for high performance microbial fuel cell. Nano Lett..

[149] Zhang Y., Sun J., Hu Y., Li S., Xu Q. (2013). Carbon nanotube-coated stainless steel mesh for enhanced oxygen reduction in biocathode microbial fuel cells. J. Power Sources.

